# Longitudinal associations of social cognition and substance use in childhood and early adolescence: findings from the Avon Longitudinal Study of Parents and Children

**DOI:** 10.1007/s00787-017-1068-x

**Published:** 2017-10-20

**Authors:** Meg E. Fluharty, Jon Heron, Marcus R. Munafò

**Affiliations:** 1 MRC Integrative Epidemiology Unit (IEU) at the University of Bristol, Bristol, UK; 2 School of Experimental Psychology, UK Centre for Tobacco and Alcohol Studies, University of Bristol, Bristol, UK; 3 School of Community and Social Medicine, University of Bristol, Bristol, UK; 4 School of Experimental Psychology, University of Bristol, 12a Priory Road, Bristol, BS8 1TU UK

**Keywords:** Social cognition, Substance use, Adolescence, Epidemiology, ALSPAC

## Abstract

Substance use is associated with impaired social cognition. Experimental studies have shown that acute intoxication of alcohol, tobacco, and cannabis decreases the performance in non-verbal, social communication and theory of mind tasks. However, in epidemiological studies the temporal direction of this association has gone relatively unstudied. We investigated both directions of association within an adolescent birth cohort: the association of social cognition with subsequent substance use, and the association of early substance use with subsequent social cognition. We used data from the Avon Longitudinal Study of Parents and Children, a UK birth cohort. Logistic regression indicated that poor childhood non-verbal communication was associated with decreased odds of adolescent alcohol (OR 0.70, 95% 0.54–0.91), tobacco (OR 0.62, 95% CI 0.47–0.83), and cannabis use (OR 0.62, 95% CI 0.46–0.83). Early adolescent substance use was associated with increased odds of poor social communication (alcohol: OR 1.46, 95% CI 0.99–2.14; tobacco: OR 1.95, 95% CI 1.33–2.86) and poor social reciprocity (alcohol: OR 1.57, 95% CI 1.18–2.09; tobacco: OR 1.92, 95% CI 1.43–2.58; cannabis: OR 1.54, 95% CI 1.16–2.05). Overall, the relationship between social cognition and substance use was different in each temporal direction. Poor non-verbal communication in childhood appeared protective against later substance use, while adolescent substance use was associated with decreased social cognitive performance.

**Electronic supplementary material:**

The online version of this article (doi:10.1007/s00787-017-1068-x) contains supplementary material, which is available to authorized users.

## Introduction

Alcohol, tobacco, and cannabis are the most commonly used substances worldwide [1–3]. In 2016, the Global Drugs Survey found that 93% of respondents reported drinking, 60% smoking tobacco, and 63% using cannabis within the past 12 months [4]. Several studies have suggested that acute administration of these substances, and/or prolonged use and abuse of these substances, is associated with deficits in social cognition (i.e., psychological processes involved in social interaction, comprising self-knowledge, perception of others, and motivational understanding). These deficits may include social (i.e., pragmatic) or non-verbal (i.e., emotion processing) communication, and/or Theory of Mind (ToM) (i.e., the ability to attribute complex mental states to others) processes, such as social reciprocity.

Studies indicate that alcohol, tobacco, and cannabis may disrupt non-verbal communication: acute intoxication from alcohol is associated with decreased reactivity to threat cues [5], while alcohol-dependent individuals display reduced accuracy in judging sadness and disgust, and require greater emotional intensity to detect fear and anger [6]. These impairments persist when alcohol-dependent individuals are detoxified [7], and can be sustained up to 2 months into sobriety [8]. In daily cigarette smokers, deficits become apparent when individuals are tobacco-deprived. Acute withdrawal in smokers is associated with diminished processing of happy faces relative to neutral faces [9], and disrupted attentional bias to facial stimuli [10]. Additionally, chronic cannabis use is associated with a reduced ability to identify emotions, particularly negative emotions [11]. However, the acute effects of different cannabinoids are distinct, with ∆-9-tetrahydrocannabinol (THC) impairing affect recognition, but cannabidiol (CBD) *improving* affect recognition [12].

Experimental studies have also shown that acute intoxication with alcohol results in ToM deficits [13]. Alcohol-dependent individual display ToM deficits, as they have difficulty identifying their own mental states and that of social partners [14, 15]. While chronic cannabis users display no change in ToM task performance compared to healthy controls, when compared at the neuroanatomical level they show differential network activation. Heavy cannabis users display less activation in the left parahippocampal gyrus, right precuneus and cuneus, but greater activation in the left cuneus and right anterior cingulate gyrus, suggesting changes at the physiological level [16]. This indicates an aberrant or greater activity of ToM network, and similar changes have been observed in at-risk psychosis populations [16, 17]. Long-term cannabinoid exposure may result in changes and functionality of the endocannabinoid system, and subsequent desensitisation of CB_1_ receptors may explain the compensatory elevated CB_1_ receptors elsewhere in the striatum [18] noted in heavy cannabis users compared to controls [19].

However, it remains unclear whether it is substance use itself causing these deficits, or whether these deficits lead to substance use (for example, to enhance certain aspects of social cognition). One argument for the latter is that children that have received social-cognitive interventions within schools and the home have lower rates of substance abuse in adolescence [20, 21]. It is also possible that the relationship between substance use and social cognition may be due to shared risk factors (genetic or environmental).

The relationship between substance use and social cognition is therefore complex, as some deficits occur rapidly with intoxication while others may arise only after longer periods of use. Furthermore, despite evidence of associations of poor social cognition with substance use, there has been relatively little research into the temporal relationships between the two to date. As individuals are most likely to experiment and initiate substance use during their adolescent period [22–24], and several studies have suggested social cognitive problems among hardened users, it is important to further understand whether substance use in early adolescence is associated with later social cognitive deficits, or whether poor social cognition in childhood is associated with later substance use.

This study, conducted using data from the Avon Longitudinal Study of Parents and Children (ALSPC), investigated the temporal associations between poor social cognitive function (non-verbal communication, social communication, and social reciprocity) and substance use behaviours (current, frequent, and age of onset). We examined the association of poor childhood social cognition with subsequent adolescent substance use, and the association of early substance use behaviour with subsequent social cognition. We hypothesised that there would be associations between poor social cognition and substance use in both temporal directions.

## Methods

### Participants

The Avon Longitudinal Study of Parents and Children (ALSPAC) is a prospective, population-based birth cohort study that recruited 14,541 pregnant women resident in Avon, UK, with expected delivery dates from April 1st 1991 to December 31st 1992 (http://www.alspac.bris.ac.uk). Information has been collected on the participants and their offspring from over 60 questionnaires and 9 clinic assessments [25]. The study website contains details of all the data that is available through a fully searchable data dictionary (http://www.bris.ac.uk/alspac/researchers/data-access/data-dictionary/). The study included 13,617 mother–offspring pairs from singleton live births who survived to at least 1 year; only these are considered here. Ethics approval for the study was obtained from the ALSPAC Ethics and Law Committee and the Local Research Ethics Committee.

**Fig. 1:**
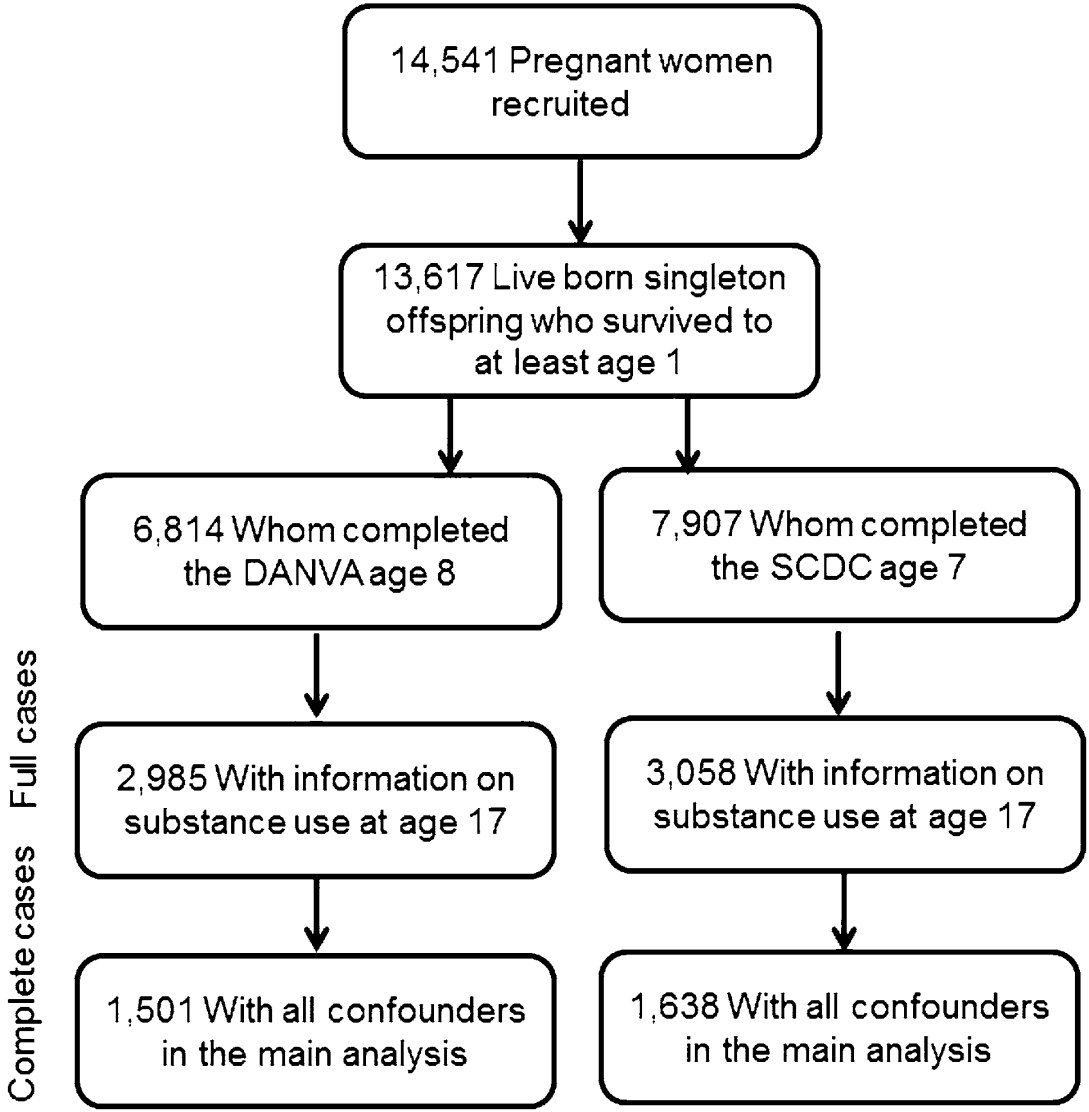
Flow diagram of final sample size in analysis of childhood social cognition (age 7/8) predicting adolescent substance use (age 18)

**Fig. 2:**
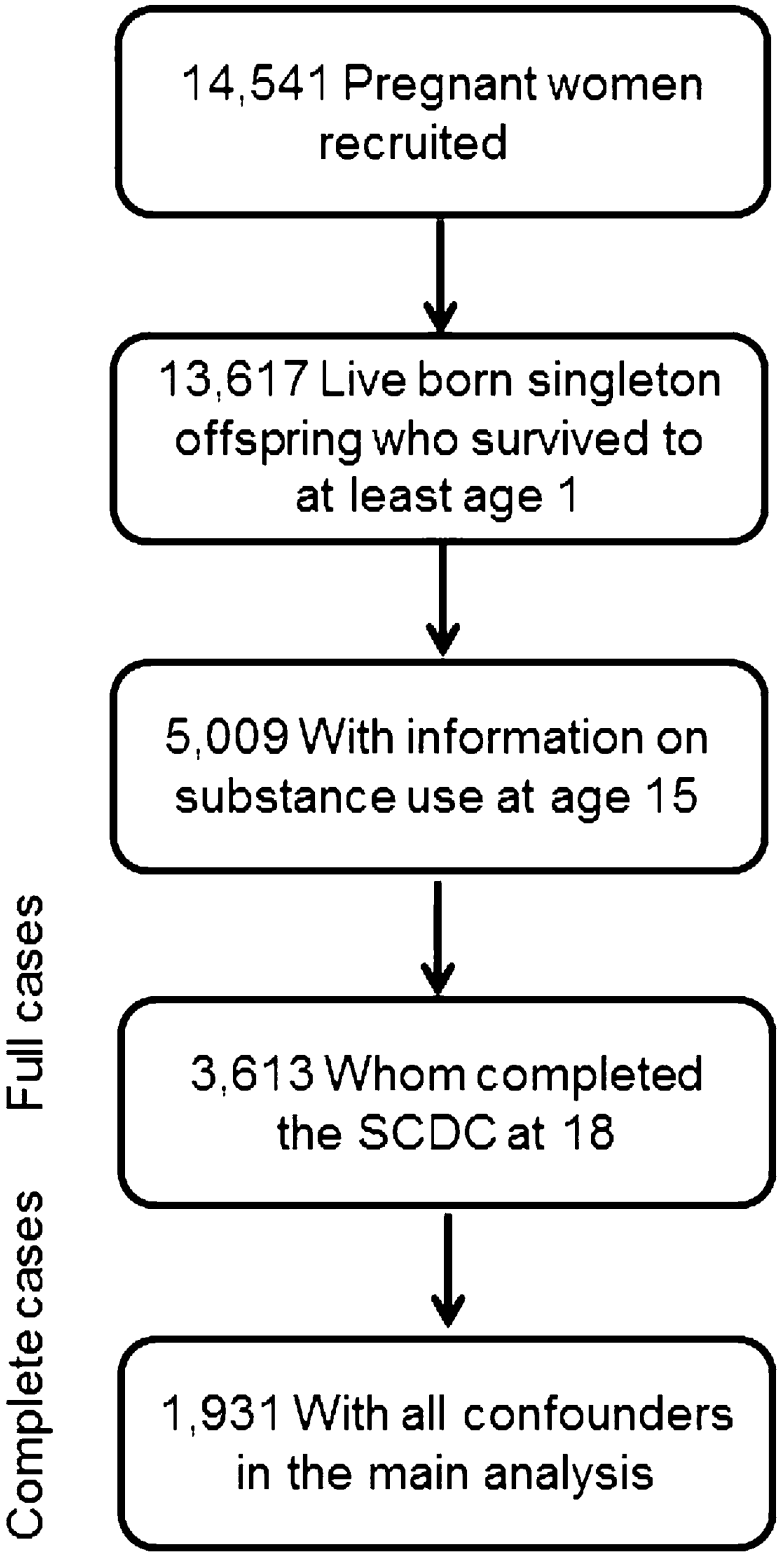
Flow diagram of final sample size in analysis of adolescent substance (age 15) use predicting social cognition (age 18)

The analysis of the association between childhood social cognition and subsequent substance use was restricted to the offspring of parents who had completed the Social and Communication Disorders Checklist (SCDC) (*N* = 3,007), SCDC sub-scale (*N* = 3,058) at age 7, and/or offspring who had completed the Diagnostic Assessment of Non-Verbal Accuracy (DANVA) (*N* = 2,985) at age 8, and offspring who had taken part in the substance use computer task at age 18 (*N* = 3,820). The analysis of the association between early adolescent substance and subsequent social cognition was further restricted to the offspring who had taken part in the substance use computer task (*N* = 5,009) at age 15, and offspring whose parents had completed the Social and Communication Disorders Checklist (SCDC) (*N* = 5,506) at age 17. Flow diagrams (Figs. 1 and 2) display the final sample size for each temporal association analysis (see Supplementary Figure 1 for a longitudinal representation of assessments).

### Measures

#### Social cognition

Non-verbal communication at age 8 was measured via computer session during a clinic visit using the faces subset of the DANVA [26]. This contains 24 photographs of children’s faces displaying an either high or low intensity version of the following emotions: happy, sad, fear, or anger. Each photograph was displayed to the children for 2 s and they responded as to what emotion they perceived. Scoring ≥ 7 total errors on the DANVA was coded as poor performance [26]. Social communication was measured by maternal completion of SCDC at offspring age 7 and 17 via questionnaire, scoring ≥ 8 out of a possible of 24 was coded as poor performance [27]. Social reciprocity at age 7 and 17 was derived from five questions on the SCDC that were specifically designed to measure social reciprocity [28, 29]. Responses of yes to ≥ three questions were coded as poor performance.

#### Substance use

Current use of alcohol, tobacco, and cannabis at age 15 was collected via computer session during a clinic visit. Individuals were classified as either current or non-users of each substance. Individuals reporting ≥ 20 drinks in the past 6 months, smoking cigarettes in the past 30 days, or using cannabis in the past 12 months were classified as current users of each respective substance. Additionally, age 18 measures of alcohol, tobacco and cannabis use were collected via a computer-based assessment during a clinic visit. Individuals were classified as users of each substance, and a user of all three substances if appropriate. Individuals scoring ≥ 8 on the Alcohol Use Disorders Identification Test (AUDIT), smoking cigarettes in the past 30 days, or using cannabis in the past 12 months were classified as users of each respective substance. Due to widespread acceptance of alcohol use in the UK, the alcohol use variable was restricted to hazardous use on the AUDIT rather than an ever/never response, as never drinkers may differ in regards to other societal factors comparable to social drinkers (e.g., abstainers for religious reasons [30, 31], or individuals with high anxiety [32]). First, individuals using all three substances were additionally classified as multi-substance users, while individuals using one to two substances were classified as non multi-substance users. Second, frequency of use was categorised as either non-weekly or weekly use. Finally, age of onset was a categorical measure based on self-reported first use of each respective substance.

#### Confounders

Based on the literature, risk factors for poor social cognition and substance use were considered as potential confounders. These included: (1) pre-birth/demographic confounders (sex [33, 34], parity [35, 36] and socioeconomic measures [37–42] including maternal social class, maternal education status, maternal home ownership status, and maternal age) as measured by baseline maternal questionnaire; (2) maternal substance use [43, 44] confounders (maternal cannabis use at offspring age 9, maternal binge drinking and smoking at offspring age 12) collected via maternal questionnaire at offspring ages 9 and/or 12; (3) childhood confounders (IQ [34, 45] measured by the Wechsler Intelligence Scale for Children-III [46], victimisation [47–49] measured by a modified version of the Bullying and Friendship Interview Schedule [50], borderline personality [51, 52] measured via interview, and peer problems [53] measured via interview, and The Strengths and Difficulties Questionnaire [54]) all collected via clinic assessment at age 8 or maternal questionnaire. Additionally, for the analysis of early substance use and subsequent social cognition, confounders included (4) previous incidence of poor social cognition (age 7 SCDC and SCDC sub-scale scores, as described above).

### Statistical Analysis

First, we examined the association of social cognition at age 7/8 (exposure) with subsequent substance use behaviour at age 18 (outcome). Next, we examined the association of early substance use behaviours at age 15 (exposure) with subsequent social cognition at age 17 (outcome). We assessed both temporal relationships before and after adjustment for covariates using logistic regression. We examined the impact of confounding by comparing unadjusted results with those adjusted for pre-birth/demographics confounders (model 1), and then additionally and cumulatively maternal substance use (model 2), childhood confounders (model 3), and (for the association of early adolescent substance use with subsequent social cognition) history of social cognition at age 7/8 (model 4). Finally, we ran a second set of confounder-adjusted analyses only including the complete cases from model 3 (for the association of childhood social cognition with subsequent substance use) or 4 (for the association of early adolescent substance use with subsequent social cognition). Both analyses were conducted unstratified and stratified by sex. Each analysis was conducted in full (total sample) and complete cases (sample restricted to data available at both time-points). Analyses were conducted in Stata version 13 (Stata Corp LP, College Station TX USA).

### Secondary analysis

Additionally, a secondary analysis was conducted after initial investigation of the DANVA exposure results. This followed the same statistical procedure as above but investigated response accuracy to individual emotions (happy, sad, fear anger) and level of affect intensity (low to high) of emotions as opposed to task accuracy as a whole.

## Results

### Characteristics of participants

Data were available on *N* = 3058 participants for the analysis of childhood social cognition with subsequent substance use, and *N* = 3613 for the analysis of early adolescent substance use with subsequent social cognition. Characteristics of these participants are shown in Table 1. Confounder characteristics and associations with each outcome are presented in Supplementary Table S1. The results presented below are from the fully adjusted models. Unadjusted and partially adjusted models are presented in Supplementary Tables (S2–S4). In general, sex-stratified analyses did not indicate any clear differences in the strength of association observed for males and females separately. The results are therefore presented unstratified, except where indicated, with sex-stratified analyses presented in Supplementary Tables S5–S8.

**Table 1:** Characteristics of participants

	*N*	Normal	Poor
Childhood social cognitive ability (age 7/8)^a,b,c^			
Social communication	7907	90% (7138)	10% (6814)
Social reciprocity	8058	84% (6757)	16% (1301)
Non-verbal communication	6814	78% (5290)	22% (1524)

	*N*	Normal	Poor
Adolescent social cognitive ability (age 18)^a,b^			
Social communication	5468	88% (4833)	12% (4300)
Social reciprocity	5571	77% (4300)	23% (1271)

	Current use		
	*N*	No	Yes
Early adolescent substance use (age 15)^d,e,f^			
Cannabis	5048	81% (4064)	19% (984)
Tobacco	5107	83% (4214)	17% (893)
Alcohol	5051	81% (4077)	19% (974)

	Current use			Frequency		
	*N*	No	Yes	*N*	≥ Weekly	< Weekly
Late adolescent substance use (age 18)^d,f,g,h,i^						
Cannabis	3820	70% (2656)	30% (1164)	1187	85% (1014)	15% (173)
Tobacco	3820	71% (2702)	29% (1118)	1181	61% (716)	39% (465)
Alcohol	3820	57% (2196)	43% (1624)	3886	74% (2874)	25% (1012)
Multi-substance	3820	86% (3268)	14% (552)			

Age	*N*	Cannabis	Tobacco	Alcohol
Age of first substance^j^				
Six	1443	0% (0)	0.10% (1)	0.20% (3)
Seven	1443	0% (0)	0.14% (2)	0.69% (10)
Eight	1443	0.10% (1)	0.30% (4)	0.90% (13)
Nine	1443	0.14% (2)	0.50% (7)	1% (21)
Ten	1443	0.14% (2)	2% (21)	6% (81)
Eleven	1443	1% (16)	5% (70)	7% (96)
Twelve	1443	4% (51)	11% (160)	17% (250)
Thirteen	1443	9% (133)	17% (246)	23% (335)
Fourteen	1443	17% (246)	21% (307)	25% (354)
Fifteen	1443	24% (345)	20% (293)	15% (212)
Sixteen	1443	31% (447)	17% (242)	4% (60)
Seventeen	1443	12% (447)	6% (81%)	0.50% (8)
Eighteen	1443	1% (19)	0.60% (8)	0% (0)
Nineteen	1443	0.14% (2)	0.10% (1)	0% (0)

^a^Poor social communication: total score of ≥ 16 on the SCDC

^b^Poor social reciprocity: scoring yes on ≥ 3 from 5 sub questions on social reciprocity on the SCDC

^c^Poor non-verbal communication: ≥ 7 total errors on the DANVA

^d^Current tobacco use: use of tobacco is past 30 days

^e^Current alcohol use: ≥ 20 drinks in past 6 months

^f^Current cannabis use: use of cannabis in past 12 months

^g^Current alcohol use: ≥ 8 AUDIT

^h^Multi-substance users were classified as being current users of all three substances

^i^Frequency of use: measure of less or more than weekly use

^j^Age of first use: categorical age of first use as measured by computerised interview

### Association of childhood social cognition (age 7/8) with adolescent substance use (age 18)

#### Non-verbal communication

Poor non-verbal communication was associated with moderately decreased odds of alcohol (fully adjusted OR 0.70, 95% CI 0.54–0.91, *P* = 0.007), tobacco (fully adjusted OR 0.62, 95% CI 0.47–0.83, *P* = 0.001), and cannabis use (fully adjusted OR 0.62, 95% CI 0.46–0.83, *P* = 0.001). These results are shown in Table 2. No clear evidence of association was observed for age of onset, or frequency of use (non-weekly/weekly) at age 18 (see Supplementary Tables S2–S3).

**Table 2:** Associations of poor childhood social cognition (age 7/8) with adolescent current substance use (age 18)

	Unadjusted				Adjustment 1 pre-birth/demographic				Adjustment 2 maternal				Adjustment 3 offspring			
	N	*OR*	*95% CI*	*p*	N	*OR*	*95% CI*	*p*	N	*OR*	*95% CI*	*p*	N	*OR*	*95% CI*	*p*
Full case analysis																
Poor non-verbal communication																
Alcohol	2985	0.74	(0.62–0.89)	0.001	2395	0.74	(0.61–0.91)	0.004	1890	0.77	(0.62–0.97)	0.027	1567	0.70	(0.54–0.91)	0.007
Tobacco	2985	0.83	(0.68–1.01)	0.059	2395	0.77	(0.62–0.97)	0.024	1890	0.70	(0.54–0.92)	0.009	1567	0.62	(0.47–0.83)	0.001
Cannabis	2985	0.72	(0.59–0.88)	0.001	2395	0.75	(0.60–0.93)	0.009	1890	0.72	(0.56–0.92)	0.010	1567	0.62	(0.46–0.83)	0.001
Multi-substance	2985	0.73	(0.56–0.96)	0.024	2395	0.75	(0.56–1.01)	0.060	1890	0.80	(0.57–1.12)	0.191	1567	0.67	(0.46–1.00)	0.047
Poor social communication																
Alcohol	3007	1.33	(1.01–1.77)	0.045	2543	1.27	(0.93–1.73)	0.126	2025	1.29	(0.91–1.84)	0.155	1609	1.42	(0.95–2.12)	0.089
Tobacco	3007	1.39	(1.03–1.87)	0.030	2543	1.32	(0.95–1.82)	0.100	2025	1.09	(0.74–1.61)	0.667	1609	1.09	(0.70–1.70)	0.708
Cannabis	3007	1.57	(1.17–2.09)	0.002	2543	1.38	(1.00–1.91)	0.047	2025	1.32	(0.91–1.91)	0.146	1609	1.56	(1.02–2.37)	0.039
Multi-substance	3007	1.55	(1.08–2.22)	0.017	2543	1.27	(0.93–1.73)	0.126	2025	1.18	(0.73–1.90)	0.508	1609	1.30	(0.73–2.20)	0.394
Poor social reciprocity																
Alcohol	3058	1.11	(0.91–1.37)	0.305	2586	1.12	(0.90–1.41)	0.304	2061	1.06	(0.82–1.38)	0.640	1638	1.10	(0.82–1.47)	0.544
Tobacco	3058	1.23	(0.99–1.53)	0.064	2586	1.21	(0.95–1.54)	0.120	2061	1.17	(0.89–1.56)	0.263	1638	1.14	(0.83 to1.58)	0.409
Cannabis	3058	1.27	(1.02–1.58)	0.029	2586	1.28	(1.01–1.62)	0.043	2061	1.29	(0.98–1.69)	0.067	1638	1.29	(0.94–1.77)	0.109
Multi-substance	3058	1.24	(0.94–1.64)	0.126	2586	1.19	(0.88–1.62)	0.256	2061	1.09	(0.76–1.56)	0.641	1638	1.02	(0.67–1.55)	0.923
Complete case analysis																
Poor non-verbal communication																
Alcohol	1567	0.68	(0.53–0.88)	0.003	1567	0.66	(0.52–0.86)	0.002	1567	0.58	(0.44–0.78)	<0.001	1567	0.70	(0.54–0.91)	0.007
Tobacco	1567	0.66	(0.49–0.88)	0.004	1567	0.64	(0.48–0.86)	0.003	1567	0.65	(0.48–0.87)	0.004	1567	0.62	(0.47–0.83)	0.001
Cannabis	1567	0.63	(0.48–0.83)	0.001	1567	0.58	(0.44–0.78)	<0.001	1567	0.58	(0.44–0.78)	<0.001	1567	0.62	(0.46–0.83)	0.001
Multi-substance	1567	0.68	(0.47–1.00)	0.050	1567	0.64	(0.44–0.95)	0.025	1567	0.66	(0.45–0.97)	0.035	1567	0.67	(0.46–1.00)	0.047
Poor social communication																
Alcohol	1609	1.28	(0.87–1.89)	0.212	1609	1.26	(0.85–1.86)	0.245	1609	1.27	(0.96–1.89)	0.228	1609	1.42	(0.95–2.12)	0.089
Tobacco	1609	1.10	(0.72–1.69)	0.648	1609	1.13	(0.73–1.74)	0.581	1609	1.14	(0.73–1.76)	0.567	1609	1.09	(0.70–1.70)	0.708
Cannabis	1609	1.47	(0.99–2.19)	0.059	1609	1.36	(0.90–2.03)	0.141	1609	1.35	(0.90–2.04)	0.147	1609	1.56	(1.02–2.37)	0.039
Multi-substance	1609	1.22	(0.72–2.07)	0.457	1609	1.20	(0.71–2.05)	0.496	1609	1.22	(0.71–2.08)	0.468	1609	1.30	(0.73–2.20)	0.394
Poor social reciprocity																
Alcohol	1638	1.07	(0.80–1.43)	0.637	1638	1.05	(0.79–1.40)	0.730	1638	1.03	(0.77–1.38)	0.827	1638	1.10	(0.82–1.47)	0.544
Tobacco	1638	1.18	(0.86–1.61)	0.311	1638	1.20	(0.87–1.64)	0.262	1638	1.17	(0.85–1.62)	0.321	1638	1.14	(0.83 to1.58)	0.409
Cannabis	1638	1.28	(0.95–1.73)	0.102	1638	1.23	(0.90–1.66)	0.188	1638	1.19	(0.87–1.62)	0.267	1638	1.29	(0.94–1.77)	0.109
Multi-substance	1638	1.04	(0.69–1.59)	0.867	1638	1.01	(0.67–1.53)	0.945	1638	1.00	(0.66–1.51)	0.984	1638	1.02	(0.67–1.55)	0.923

Poor non-verbal communication: ≥ seven total errors on the DANVA

Poor social reciprocity: scoring yes on ≥ three from five sub questions on social reciprocity on the SCDC

Poor social communication: total score of ≥ 16 on the SCDC

Current alcohol use: ≥ 8 AUDIT

Current tobacco use: use of tobacco is past 30 days

Current cannabis use: use of cannabis in past 12 months

Multi-substance users were classified as being current users of all three substances. (1) Adjusted for pre-birth/demographic characteristics: sex, parity, maternal social class, home ownership status, and maternal age. (2) Additionally adjusted for maternal substance use confounders: maternal cannabis use (offspring age 9) maternal smoking and binge drinking (offspring age 12). (3) Additionally adjusted for offspring confounders: IQ, peer problems, victimisation, borderline personality diagnosis

#### Social communication and social reciprocity

There was no clear evidence of an association of either poor social communication or social reciprocity with alcohol, tobacco, cannabis, or all substance use. These results are shown in Table 2. Additionally, no clear evidence of association was observed for age of onset, or frequency of use (non-weekly/weekly) at age 18 (see Supplementary Tables S2–S3).

#### Secondary analyses

To further investigate the association of non-verbal communication and current substance use, we investigated the DANVA by individual emotion and intensity. There was no clear pattern of association across the individual emotions (see Supplementary Table S4). However, individuals displaying reduced ability to identify emotions in general, as demonstrated by poor identification of both ‘low’ and ‘high’ intensity emotionally expressive faces, had decreased odds of substance use onset, similar to the results seen above. Poor identification of low and high intensity faces was associated with decreased odds of alcohol, tobacco, and cannabis use, and this was robust to adjustment (see Table 3 for details).

**Table 3:** Associations of poor childhood (age 7/8) identification of high and low intensity faces with adolescent current substance use (age 15)

	Unadjusted				Adjustment 1 pre-birth/demographic				Adjustment 2 maternal				Adjustment 3 offspring			
	N	*OR*	*95% CI*	*p*	N	*OR*	*95% CI*	*p*	N	*OR*	*95% CI*	*p*	N	*OR*	*95% CI*	*p*
Full case analysis																
Poor identification of high intensity faces																
Alcohol	2985	0.75	(0.62–0.91)	0.004	2398	0.78	(0.63–0.96)	0.017	1890	0.77	(0.60–0.98)	0.032	1567	0.70	(0.53–0.92)	0.010
Tobacco	2985	0.75	(0.60–0.93)	0.007	2398	0.76	(0.69–0.96)	0.020	1890	0.72	(0.55–0.96)	0.023	1567	0.63	(0.46–0.87)	0.004
Cannabis	2985	0.69	(0.56–0.85)	0.001	2398	0.73	(0.58–0.92)	0.009	1890	0.69	(0.53–0.91)	0.008	1567	0.63	(0.46–0.85)	0.003
Multi-substance	2985	0.62	(0.46–0.83)	0.002	2398	0.66	(0.48–0.92)	0.012	1890	0.71	(0.49–1.02)	0.064	1567	0.61	(0.39–0.93)	0.230
Poor identification of low intensity faces																
Alcohol	2985	0.72	(0.60–0.87)	0.001	2398	0.68	(0.55–0.84)	<0.001	1890	0.68	(0.53–0.85)	0.001	1567	0.67	(0.51–0.85)	0.002
Tobacco	2985	0.92	(0.75–1.12)	0.418	2398	0.82	(0.66–1.03)	0.091	1890	0.73	(0.56–0.96)	0.023	1567	0.67	(0.50–0.90)	0.009
Cannabis	2985	0.83	(0.68–1.01)	0.063	2398	0.78	(0.62–0.97)	0.027	1890	0.73	(0.57–0.95)	0.017	1567	0.68	(0.51–0.90)	0.008
Multi-substance	2985	0.86	(0.66–1.12)	0.254	2398	0.84	(0.62–1.13)	0.244	1890	0.79	(0.57–1.12)	0.185	1567	0.73	(0.49–1.07)	0.108
Complete case analysis																
Poor identification of high intensity faces																
Alcohol	1567	0.68	(0.53–0.89)	0.005	1567	0.67	(0.51–0.87)	0.003	1567	0.68	(0.52–0.89)	0.005	1567	0.70	(0.53–0.92)	0.010
Tobacco	1567	0.64	(0.47–0.88)	0.005	1567	0.64	(0.47–0.88)	0.005	1567	0.66	(0.48–.90)	0.009	1567	0.63	(0.46–0.87)	0.004
Cannabis	1567	0.63	(0.47–0.85)	0.002	1567	0.59	(0.44–0.80)	0.001	1567	0.61	(0.45–0.83)	0.001	1567	0.63	(0.46–0.85)	0.003
Multi-substance	1567	0.60	(0.40–0.92)	0.018	1567	0.58	(0.38–0.89)	0.012	1567	0.60	(0.40–0.93)	0.021	1567	0.61	(0.39–0.93)	0.230
Poor identification of low intensity faces																
Alcohol	1567	0.66	(0.52–0.86)	0.002	1567	0.64	(0.50–0.83)	0.001	1567	0.65	(0.50–0.84)	0.001	1567	0.67	(0.51–0.85)	0.002
Tobacco	1567	0.70	(0.53–0.94)	0.018	1567	0.68	(0.51–0.92)	0.011	1567	0.69	(0.51–0.92)	0.013	1567	0.67	(0.50–0.90)	0.009
Cannabis	1567	0.67	(0.51–0.89)	0.006	1567	0.68	(0.51–0.90)	0.007	1567	0.66	(0.50–0.88)	0.005	1567	0.68	(0.51–0.90)	0.008
Multi-substance	1567	0.76	(0.52–1.10)	0.144	1567	0.72	(0.49–1.05)	0.088	1567	0.72	(0.49–1.06)	0.100	1567	0.73	(0.49–1.07)	0.108

Poor identification of high intensity faces: ≥ three errors on high intensity DANVA faces

Poor identification of low intensity faces: ≥ five errors on low intensity DANVA faces

Current alcohol use: ≥ eight AUDIT

Current tobacco use: use of tobacco is past 30 days

Current cannabis use: use of cannabis in past 12 months

Multi-substance users were classified as being current users of all three substances. (1) Adjusted for pre-birth/demographic characteristics: sex, parity, maternal social class, home ownership status, and maternal age. (2) Additionally adjusted for maternal substance use confounders: maternal cannabis use (offspring age 9) maternal smoking and binge drinking (offspring age 12). (3) Additionally adjusted for offspring confounders: IQ, peer problems, victimisation, borderline personality diagnosis

### Association of early adolescent substance use (age 15) with later social cognition (age 18)

#### Social communication

Increased odds of poor social communication was associated with earlier adolescent alcohol (fully adjusted OR 1.46, 95% CI 0.99–2.14, *P* = 0.051), and tobacco use (fully adjusted OR 1.95, 95% CI 1.33–2.86, *P* = 0.001). There was no clear evidence of an association of poor social communication with earlier cannabis use. These results are shown in Table 4. In stratified analyses, associations were slightly stronger for males, with respect to tobacco outcomes.

**Table 4:** Association of current substance use (age 15) predicting poor social cognition (age 18)

	Unadjusted				Adjustment 1 pre-birth/demographic				Adjustment 2 maternal				Adjustment 3 offspring				Adjustment 4 poor social cognition			
	N	*OR*	*95% CI*	*p*	N	*OR*	*95% CI*	*p*	N	*OR*	*95% CI*	*p*	N	*OR*	*95% CI*	*p*	N	*OR*	*95% CI*	*p*
Full case analysis																				
Alcohol																				
Social communication	3631	1.21	(0.94–1.57)	0.139	3089	1.45	(1.10–1.91)	0.009	2550	1.38	(1.00–1.88)	0.048	2002	1.49	(1.03–2.14)	0.033	1915	1.46	(0.99–2.14)	0.051
Social Reciprocity	3704	1.36	(1.12–1.65)	0.002	3147	1.50	(1.22–1.86)	<0.001	2599	1.40	(1.11–1.78)	0.005	2041	1.59	(1.21–2.08)	0.001	1976	1.57	(1.18–2.09)	0.002
Tobacco																				
Social communication	3662	2.05	(1.61–2.61)	<0.001	3113	2.03	(1.55–2.68)	<0.001	2570	2.02	(1.48–2.77)	<0.001	2020	1.97	(1.37–2.84)	<0.001	1933	1.95	(1.33–2.86)	0.001
Social Reciprocity	3736	1.97	(1.63–2.39)	<0.001	3172	1.06	(1.66–2.55)	<0.001	2620	2.02	(1.58–2.57)	<0.001	2059	1.98	(1.49–2.62)	<0.001	1994	1.92	(1.43–2.58)	<0.001
Cannabis																				
Social communication	3637	1.38	(1.07–1.77)	0.012	3095	1.31	(0.98–1.73)	0.064	2555	1.28	(0.39–1.77)	0.128	2009	1.32	(0.91–1.91)	0.146	1992	1.26	(0.85–1.86)	0.255
Social Reciprocity	3710	1.54	(1.27–1.86)	<0.001	3153	1.54	(1.25–1.90)	<0.001	2604	1.55	(1.22–1.96)	<0.001	2048	1.57	(1.19–2.06)	0.001	1983	1.54	(1.16–2.05)	0.003
Complete case analysis																				
Alcohol																				
Social communication	1915	1.30	(0.91–1.87)	0.151	1915	1.38	(0.96–1.98)	0.087	1915	1.41	(0.98–2.04)	0.660	1915	1.49	(1.02–2.15)	0.037	1915	1.46	(0.99–2.14)	0.051
Social Reciprocity	1976	1.44	(1.10–1.88)	0.008	1976	1.53	(1.17–2.01)	0.002	1976	1.54	(1.17–2.02)	0.002	1976	1.61	(1.22–2.13)	0.001	1976	1.57	(1.18–2.09)	0.002
Tobacco																				
Social communication	1933	1.95	(1.36–2.78)	<0.001	1933	1.94	(1.34–2.79)	<0.001	1933	1.95	(1.34–2.82)	<0.001	1933	1.94	(1.34–2.82)	<0.001	1933	1.95	(1.33–2.86)	0.001
Social Reciprocity	1994	1.92	(1.46–2.53)	<0.001	1994	1.99	(1.50–2.64)	<0.001	1994	1.97	(1.49–2.62)	<0.001	1994	2.00	(1.50–2.66)	<0.001	1994	1.92	(1.43–2.58)	<0.001
Cannabis																				
Social communication	1922	1.16	(0.89–1.67)	0.440	1922	1.16	(0.80–1.68)	0.438	1922	1.20	(0.82–1.75)	0.344	1922	1.27	(0.86–1.85)	0.227	1992	1.26	(0.85–1.86)	0.255
Social Reciprocity	1983	1.44	(1.11–1.88)	0.007	1983	1.48	(1.13–1.93)	0.004	1983	1.48	(1.13–1.95)	0.005	1983	1.57	(1.19–2.06)	0.001	1983	1.54	(1.16–2.05)	0.003

Poor social reciprocity: scoring yes on ≥ three from five sub questions on social reciprocity on the SCDC

Poor social communication: total score of ≥ 16 on the SCDC

Current alcohol use: ≥ 20 drinks in past 6 months

Current tobacco use: use of tobacco is past 30 days

Current cannabis use: use of cannabis in past 12 months. (1) Adjusted for pre-birth/demographic characteristics: sex, parity, maternal social class, home ownership status, and maternal age. (2) Additionally adjusted for maternal substance use confounders: maternal cannabis use (offspring age 9) maternal smoking and binge drinking (offspring age 12). (3) Additionally adjusted for offspring confounders: IQ, peer problems, victimisation, borderline personality diagnosis. (4) Additionally adjusted for poor SCDC or SCDC sub-scale (respectively) at offspring age 7

#### Social reciprocity

Increased odds of poor social reciprocity was associated with earlier adolescent alcohol (fully adjusted OR 1.57, 95% CI 1.18–2.09, *P* = 0.002), tobacco (fully adjusted OR 1.92, 95% CI 1.43–2.58, *P* = < 0.001), and cannabis use (fully adjusted OR 1.54, 95% CI 1.16–2.05, *P* = 0.003). These results are shown in Table 4. In stratified analyses, associations were slightly stronger for males, with respect to tobacco outcomes (see Supplementary Tables S8).

## Discussion

Our results indicate that, in this cohort, poor non-verbal communication at age 8 is associated with *decreased* alcohol, tobacco, and cannabis use. Adjustment for pre-birth/demographic, maternal, and childhood confounders strengthened the associations for tobacco and cannabis use, but weakened the associations for alcohol. We analysed individual emotions within the DANVA to identify whether sensitivity to specific emotions were driving this association. No pattern of association was found for individual emotions, although poor identification of both low and high intensity of emotional expression was associated with alcohol, tobacco, cannabis, and all substance use. Adjustment for confounders strengthened the associations for alcohol, tobacco, and cannabis, but weakened the association for all substance use. Interestingly, poor non-verbal communication appeared to be protective against later substance use; thus the deficits in non-verbal communication previously reported in substance users are more likely to be the outcome of prolonged use [6–8, 10, 11], as opposed to reflecting self-medication of these deficits. In the opposite temporal direction, our results indicate that current alcohol, tobacco, and/or cannabis use at age 15 is associated with poor social communication and social reciprocity at 17. In all cases, adjustment for pre-birth, maternal, childhood, or previous indication of poor social cognition (age 7) did not substantially alter these associations. As both analyses adjust for previous indication of poor social cognition prior to the onset of any substance use (age 7), this suggests that being a current user of alcohol, tobacco, and cannabis may have a substantial impact on social cognitive abilities.

Generally, these analyses suggest that social cognitive deficits may result from the initiation and/or regular use of these substances. While previous literature has suggested these social cognitive deficits can arise during periods of acute intoxication [5, 12] or withdrawal [10], our results suggest these deficits remain present over longer periods of time among users. Alcohol dependence has been associated with impaired semantic memory (i.e., deficits general knowledge accumulated through personal experience). As semantic memory may be necessary for the maintenance of social networks [55], this may subsequently lead to more specific social cognitive deficits [14]. Prolonged nicotine exposure may dysregulate the hypothalamic–pituitary–adrenal system, resulting in the hypersecretions of cortisol and alterations in the activity of the associated monoamine neurotransmitter system, which contributes to stress-regulation [56]. This may result in individuals being more susceptible to environmental stressors and associated difficulties with affect and emotional regulation [57, 58]. Finally, evidence from imaging studies indicate neuroanatomical changes in heavy cannabis uses associated with prolonged endocannabinoid exposure, and the subsequent desensitisation of CB_1_ receptors in the brain, requiring compensatory CB1 receptor activity elsewhere in the striatum [16–19]. Previous literature indicates strong familial bonds and open communication within families and schools may serve as a protective factor, or help to delay adolescent substance initiation [59–62]. However, in the other temporal direction (i.e., poor social cognition and subsequent substance use), there is currently little evidence. Our analyses help to rule out the possibility of reverse causality, and strengthen our findings that substance use is associated with later impaired social cognition. Additionally, this analysis suggested that poor non-verbal communication may in fact be *protective* with respect to subsequent substance use. While this is clearly an area that warrants additional research and replication, one possible explanation for this finding is that adolescents with poor emotion recognition skills may less likely to have larger social groups [63, 64] and therefore less likely to engage in substance use due to less social inclusion [65–68].

Strengths of this study include a rich data set with multiple social cognitive and substance use variables collected at several time-points throughout the adolescence and early adulthood. This allows for the analysis of both temporal directions as well as examining different facets of social cognition. Additionally, a robust approach was taken to minimise confounding, using a range of possible confounders from pre-birth throughout adolescence. There are also some limitations in our study to consider. First, some of our exposures were self-reported by the child (DANVA) while others were parent-completed (SCDC and DAWBA). Previous studies have indicated parental rating of offspring well-being to be more positive compared to self-report by offspring [69]. Similarly, the maternal-reported measure of SCDC taken when offspring were aged 17 may be capturing a breakdown in family communication or adolescent disobedience, as opposed to social cognition, due to the generally rebellious nature of the adolescent period. However, a recent genome-wide association study conducted in ALSPAC found evidence of a genome-wide association of SCDC measures at age 17, suggesting there is a genetic architecture of social communication that can be reliably captured by the maternal SCDC measure [70]. Second, SCDC scores are known to remain constant across age groups [27], while studies have indicated DANVA scores to improve with age [26]. This is a potential problem if the ranking of scores across the population is not consistent; however, previous ALSPAC studies have indicated a test–retest reliability in the DANVA of 0.84 [71]. Third, as maternal data are collected frequently and are more extensive than partner data within ALSPAC, we only investigated the impact of maternal confounding. Fourth, our substance use outcomes are all reliant on self-report and we were not able to biochemically validate these responses. Additionally, we drew our outcomes from age 18, which provided us with a large sample size of individuals whom had ever used substances. However, there were notably fewer individuals answering questions regarding frequency of use, which may have contributed to the low power for these analyses. Fifth, it is possible that our variable for multi-substance current use simply reflects current cannabis use, since cannabis users typically also consume alcohol and tobacco [72]. Finally, there was evidence of differential loss to follow-up, as some children with high SCDC scores, were slightly more likely to drop out of the study before substance use and social cognition outcome data was obtained. However, this does not necessarily imply selection bias in the association between social cognition and later substance use [73], and comparisons of full and complete cases display little change in results due to sample size.

Overall, we found differing patterns of relationships between social cognition and substance use behaviour dependent on the specific social cognition examined and temporal direction of association. While poor non-verbal communication in childhood appeared to have protective factors against later substance use, early adolescent substance use was associated with decreased social cognitive performance. This association would be worth pursuing for replication in cohorts with similar or richer data on social cognitive variables. Furthermore, the use of differing statistical analyses and methods will additionally help to strengthen these findings. Given that causality cannot be inferred from observational data alone, future epidemiological studies investigating these associations should consider alternative statistical techniques. For example, studies can use genetic variants associated with substance use or social communication as proxies for these exposures within a Mendelian randomization framework.

## Electronic supplementary material

Below is the link to the electronic supplementary material.

Supplementary material 1 (XLSX 101 kb)
